# Year 100 of the *American Journal of Tropical Medicine and Hygiene*: A Remarkable Year

**DOI:** 10.4269/ajtmh.20-1504

**Published:** 2021-01

**Authors:** Philip J. Rosenthal

**Affiliations:** University of California, San Francisco, San Francisco, California

Two years ago, we celebrated an anniversary of sorts, with publication of volume 100 of the *American Journal of Tropical Medicine and Hygiene* (AJTMH).^1^ That milestone was a bit artificial, as it included counting volumes of our predecessor journals, with varied numbers of volumes published per year, but it offered a chance to review progress. Now we celebrate a more precise anniversary, 100 years after publication of the first issue of the *American Journal of Tropical Medicine* in 1921. That journal merged in 1952 with the Journal of the National Malaria Society to form our modern journal. Please see our prior editorial for a review of how we got here.^1^ In this report, we discuss where we are and where we are going.

Our anniversary comes at the completion of a remarkable year. Early in 2020, we were addressing the complicated politics of a rancorous election year in the U.S. Concerns about politically motivated misunderstanding and misuse of science have been widespread in 2020, leading to a journal editorial bemoaning two landmark decisions by the U.S. administration, to cancel NIH funding for an ongoing study of coronaviruses, and to withhold support for the WHO.^2^ Assaults on science have been fierce and quite astounding. In these challenging times, the AJTMH will continue to serve as a forum for the leadership of the ASTMH^2–4^ and our colleagues^5–20^ concerning issues of vital importance to the international tropical medicine and public health communities.

Within the first few months of 2020, a pandemic was upon us. Initially, Americans watched in horror as the COVID-19 pandemic wreaked havoc in Asia and then Europe, but soon the pandemic proved to be a horrific problem also in the United States and nearly every country. Unlike pandemics from the past, COVID-19 has emerged in the context of modern communication and a scientific community poised for modern investigation. This has led to an explosion of scientific investigation on SARS-CoV-2 and COVID-19. Concurrently, experts have seized the opportunity to comment on every aspect of this all-too-often controversial pandemic, from clinical medicine to epidemiology to social and behavioral matters. With these trends, there has been submission and eventual publication of a remarkably large number of manuscripts on COVID-19. On December 14, 2020, a PubMed search of “COVID-19” identified 77,328 published articles. No doubt, this represents an unprecedented profusion of publications on a single new topic. With more new science on a brand new topic than ever before, we should be in the best shape possible to tackle the pandemic. Of course, it is not so simple, as the pandemic is complex, and as politicians and their proxies continue to misread scientific results to fit their political purposes. The spread of misinformation has paralleled that of high-quality scientific data.^12,16,21–23^ But, more light is better than less, and we should keep the faith that despite setbacks, knowledge will ultimately conquer ignorance in the fight against COVID-19.

**Figure 1.**
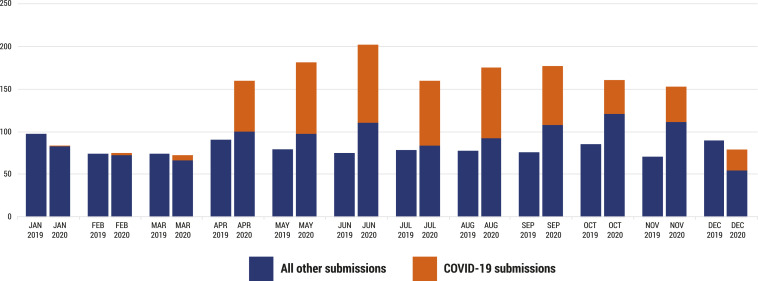
The number of manuscripts submitted to the AJTMH in each month of 2019 and 2020 is shown. Manuscripts submitted through December 14, 2020 are included.

The impact of COVID-19 on the AJTMH has been profound. After a handful of submissions related to the pandemic in the first few months of 2020, the number of submissions exploded in April (Figure 1). Soon, the journal was receiving about twice its typical volume of submissions. The excess was primarily due to articles on COVID-19. As of December 14, 2020, we have considered hundreds and published 184 articles on COVID-19, ranging from case reports and descriptive studies helping us to characterize the pandemic in its early days to early clinical trials of a range of potential therapies to perspectives addressing different aspects of COVID-19 management and policy. Submissions of non-COVID-19 manuscripts have also increased. Large numbers of submissions to the AJTMH led to a marked increase in workload for our editorial staff, our editors, and our reviewers. Happily, all have stepped up, and the journal has been able to maintain prompt reviews for most submissions (average of 24 days). Considering the urgency of the pandemic, the journal has expedited reviews of manuscripts on COVID-19 as much as possible (average of 14 days). As of this writing late in 2020, the rate of submissions on COVID-19 has slowed, but these submissions continue, and we can anticipate that however quickly the pandemic abates in the coming months, the science of this pandemic will be featured in the medical literature for years to come.

Year 100 has been a remarkable year for the AJTMH. What does the future hold? As COVID-19 slowly fades as a focus, it is hoped that some new linkages for the AJTMH will remain strong. In 2020, we have published many more papers on a range of disciplines, including clinical virology, intensive care unit medicine, and radiology, to name a few, than in past years. Although most attention in the United States has gone to COVID-19 in highly developed countries, which have been particularly hard-hit, the pandemic has also spurred the provision of advanced medical care in many low- and low-middle–income countries that typically offer such care on a very limited basis. Availability of advanced care has certainly been spotty, but we can hope that various COVID-19–inspired advances, from efficient diagnostics^17,24,25^ to point-of-care ultrasound^26–30^ and relatively low-cost means of mechanical ventilation,^31,32^ will remain in less developed countries as the pandemic disappears. Evaluation of the utilization of new technologies for the care of tropical diseases is a major focus of the AJTMH, and so advances facilitated by the pandemic will be featured. We can hope that these advances will improve care for a range of tropical diseases, offering a modest silver lining after the ravages of the pandemic.

As we reach the end of 2020, the future is hard to predict. But, it is a certainty that a great many tropical diseases will continue to afflict our planet. Changes will undoubtedly be seen over time, with some ailments diminishing; we expect continued good progress against many vector-borne diseases, a range of helminth infections, and diseases of poor sanitation. Progress remains challenging for some of our biggest killers, including HIV infection, tuberculosis, and malaria, and control of these diseases has taken a hit from the pandemic, but we can foresee getting back to the level of progress seen in recent years. Furthermore, as we learned so clearly in 2020, new infections will emerge, and noncommunicable diseases are on the rise. Through the slings and arrows of the modern world, from persistent endemic infectious diseases, to increasing non-communicable diseases, to pandemics, and political craziness, the AJTMH will remain a force for disseminating the latest information and recommendations on tropical medicine and public health.
